# Immunoprotective properties of recombinant LigA and LigB in a hamster model of acute leptospirosis

**DOI:** 10.1371/journal.pone.0180004

**Published:** 2017-07-13

**Authors:** Karen V. Evangelista, Kristel Lourdault, James Matsunaga, David A. Haake

**Affiliations:** 1 Veterans Affairs Greater Los Angeles Healthcare System, Los Angeles, California, United States of America; 2 Department of Medicine, David Geffen School of Medicine at UCLA, Los Angeles, California, United States of America; 3 Department of Urology, David Geffen School of Medicine at UCLA, Los Angeles, California, United States of America; 4 Department of Microbiology, Immunology and Molecular Genetics, UCLA, Los Angeles, California, United States of America; Instituto Butantan, BRAZIL

## Abstract

Leptospirosis is the most widespread zoonosis and is considered a major public health problem worldwide. Currently, there is no widely available vaccine against leptospirosis for use in humans. A purified, recombinant subunit vaccine that includes the last six immunoglobulin-like (Ig-like) domains of the leptospiral protein LigA (LigA7’-13) protects against lethal infection but not renal colonization after challenge by *Leptospira interrogans*. In this study, we examined whether the addition of the first seven Ig-like domains of LigB (LigB0-7) to LigA7’-13, can enhance immune protection and confer sterilizing immunity in the Golden Syrian hamster model of acute leptospirosis. Hamsters were subcutaneously immunized with soluble, recombinant LigA7’-13, LigB0-7, or a combination of LigA7’-13 and LigB0-7 in Freund’s adjuvant. Immunization with Lig proteins generated a strong humoral immune response with high titers of IgG that recognized homologous protein, and cross-reacted with the heterologous protein as assessed by ELISA. LigA7’-13 alone, or in combination with LigB0-7, protected all hamsters from intraperitoneal challenge with a lethal dose of *L*. *interrogans* serovar Copenhageni strain Fiocruz L1-130. However, bacteria were recovered from the kidneys of all animals. Of eight animals immunized with LigB0-7, only three survived *Leptospira* challenge, one of which lacked renal colonization and had antibodies to native LigB by immunoblot. In addition, sera from two of the three LigB0-7 immunized survivors cross-reacted with LigA11-13, a region of LigA that is sufficient for protection. In summary, we confirmed that LigA7’-13 protects hamsters from death but not infection, and immunization with LigB0-7, either alone or in combination with LigA7’-13, did not confer sterilizing immunity.

## Introduction

Leptospirosis is the most widespread zoonotic disease affecting both humans and animals. Infection by pathogenic strains of *Leptospira* spp. commonly occurs through direct contact with an infected animal’s urine or indirectly through contaminated water. Almost all mammals can harbor *Leptospira* in the proximal renal tubules, from which spirochetes are excreted in urine. Rats serve as a major carrier in human leptospirosis particularly in urban settings. Infection in rats is mostly asymptomatic, with bacteria cleared from the bloodstream and organs except the renal tubules leading to urinary shedding of up to 10^7^ leptospires per milliliter months after initial infection [1, 2]. Humans, on the other hand, serve as accidental hosts with whom infection is acute and occasionally fatal. Leptospirosis in humans can present as an acute, self-limited disease characterized by an abrupt onset of fever, headache, chills, nausea and vomiting, myalgia, and less commonly skin rashes. A severe disease form is Weil’s syndrome, characterized by multiorgan system complications including jaundice, acute hepatic and renal dysfunction, and hemorrhage [1–5]. An infected individual may also be asymptomatic and shed leptospires, similar to maintenance animal hosts. Asymptomatic renal colonization and urinary shedding were observed among a group of individuals in the Peruvian Amazon [6]. However, the prevalence of persistently infected individuals and their relevance to disease transmission are unknown.

Although killed, whole-cell leptospiral vaccines are available in some countries including France, Cuba, and Japan [7–9], there is currently no widely available vaccine against leptospirosis for use in humans. Most vaccines available in the market are for veterinary use and are either inactivated leptospires (heat-killed or formalin-killed) consisting of a limited panel of serovars or outer membrane fractions. Immunity is directed mainly against leptospiral lipopolysaccharide (LPS), whose structure differs among the several hundred serovars of *Leptospira*. These vaccines produce short-term immunity and do not confer cross-protection against all pathogenic serovars [10, 11]. Therefore, there is a great need for development of vaccines that elicit a robust humoral and cellular immunity against pathogenic *Leptospira* spp., providing immunoprotection across serovars and sterilizing immunity against bacterial infection.

Surface-exposed outer membrane proteins (OMPs) that play a role in bacterial virulence and can be recognized by the host immune response early during the infection are attractive vaccine candidates. They have the potential to elicit cross-protection against different serovars of pathogenic *Leptospira* [12]. A wide range of OMPs have been tested for their protective ability including LipL32 [13, 14], OmpL1 and LipL41 [15, 16], and leptospiral immunoglobulin (Ig)-like proteins A (LigA) and B (LigB) [8, 17–27].

LigA and LigB consist of a lipoprotein signal peptide, followed by a series of 12–13 Ig-like domains [28, 29] similar to those found in bacterial virulence determinants invasin in *Yersinia pseudotuberculosis* [30], and intimin in *Escherichia coli* [31]. The first six domains and a portion of the seventh domain (conserved region) are nearly identical between the two Lig proteins, with an amino acid sequence identity of 99%. LigB, unlike LigA, has a unique carboxy-terminal non-repeat domain, and is found in all pathogenic strains of *Leptospira* [28, 29]. While LigA is not found in all pathogenic species, it is expressed by most characterized strains of *L*. *interrogans*, a major human pathogen [10]. Lig proteins are leptospiral surface components, and have been shown to bind extracellular matrix (ECM) components fibronectin, elastin, tropoelastin, laminin, and collagens I, III, and IV [32–37]. Therefore, these proteins may be involved in bacterial attachment to host tissues and colonization. Additionally, heterologous expression of *ligA* and *ligB* partially protects the non-pathogen *L*. *biflexa* from killing by human serum. Cleavage of the complement components C3b and C4b by complement regulators bound by LigA and LigB is believed to be responsible for this observation [38–40]. The Lig proteins also acquire key host proteins that participate in hemostasis. They bind to plasminogen, which is converted to plasmin in the presence of urokinase-type plasminogen activator, leading to cleavage of C3b, C5, and fibrinogen [39]. LigB also suppresses coagulation by capturing fibrinogen and factor XIII [33, 41, 42]. In virulent strains, *ligA* and *ligB* are upregulated at physiological osmolarity [32, 43, 44] and temperature [45] while expression is lost when strains are culture attenuated [28]. These observations suggest that the expression of *ligA* and *ligB* during infection may facilitate bacterial survival and dissemination. While the genetic knockout of *ligB* in *L*. *interrogans* serovar Copenhageni does not affect the virulence in hamsters or kidney colonization in rats [46], the targeted repression of both *ligA* and *ligB* in *L*. *interrogans* serovar Manilae results in attenuation in hamsters and failure to recover bacteria from tissues [47]. These findings suggest the overlapping or redundant roles of LigA and LigB in pathogenesis.

A number of studies have shown that LigA subunit or DNA vaccines generated from different serovars protected rodent models against homologous challenge infection [18–21, 23–26]. For the Copenhageni serovar, a carboxy-terminal LigA fragment starting within the seventh Ig domain is protective in the hamster model [26]. Ig domains 11–12, along with 10 or 13, are sufficient for protection [18]. Our group has also demonstrated that LigA domains 7–13 expressed as a lipoprotein in live *E*. *coli* and administered orally improved the hamster’s survival following *L*. *interrogans* lethal challenge [8]. However, LigA did not confer sterilizing immunity in any of these studies; renal colonization was observed in all immunized animals.

LigB also has potential as subunit vaccine [17, 23, 27]. The *ligB* gene, unlike *ligA*, is present in all pathogenic serovars of *Leptospira* that have been examined [29, 48]. Yan *et al*. [27] showed that subcutaneous immunization with the region of LigB that is nearly identical to LigA (first seven domains) improved the survival of hamsters challenged with *L*. *interrogans* serovar Pomona with corresponding reduction in kidney, liver, and spleen lesions. Immunization with LigB0-7 also increased splenic lymphocyte proliferation and upregulated transcripts of the Th1 cytokines IL-12 and IFN-γ, suggesting activation of cell-mediated immunity. This response is thought to be necessary to prevent leptospiral renal colonization and urinary shedding in cattle vaccinated with *L*. *borgpetersenii* serovar Hardjo [49].

In the current study, we determined if addition of LigB to a LigA vaccine can confer sterilizing immunity. We cloned and expressed soluble Ig-like domains 7–13 of LigA (LigA7’-13) and the N-terminal region including Ig domains 1–7 of LigB (LigB0-7) in *E*. *coli*. Using a hamster model of acute leptospirosis, we immunized animals with the recombinant proteins alone or in combination, to assess their ability to protect the animals from fatal infection with *Leptospira*, and provide sterilizing immunity of the kidney.

## Materials and methods

### Bacterial cultures

*Leptospira interrogans* serovar Copenhageni (pathogenic, strain Fiocruz L1-130) were grown in liquid Ellinghausen-McCullough-Johnson-Harris (EMJH) medium [10, 50, 51] at 30^°^C. This strain has a median lethal dose (LD_50_) range of 37–10^4^ [52, 53], and a reported median endpoint dose (ED_50_) of ~20 [8] in hamsters challenged intraperitoneally. The genome sequence was previously reported by Nascimento *et al*. [54].

Chemically competent *E*. *coli* strain DH5α (New England Biolabs, Ipswich, MA) was used for gene cloning, while strain BLR (DE3) pLysS (EMD Millipore, Charles, MO) was used for protein expression and purification. *E*. *coli* were grown in Luria-Bertani (LB) or 2x YT medium (BD Biosciences, Sparks, MD) supplemented with 0.2% v/v dextrose and 100 μg/ml ampicillin (Sigma-Aldrich, St. Louis, MO). LB agar, containing 15 g/L agar formulation, was used for plating (Mo Bio Laboratories, Carlsbad, CA).

### Animals

The work involving animals in this study was carried out according to the regulations of, and approved by, the Institutional Animal Care and Use Committee (IACUC, Protocol 09018–14) at the Veterans Affairs Greater Los Angeles Healthcare System (VAGLAHS), Los Angeles, CA. All animal experiments were performed under Animal Biosafety Level (ABSL-2) conditions. Female Golden Syrian hamsters (strain HsdHan:AURA) were purchased at the age of 3–4 weeks from Envigo RMS (Indianapolis, IN). The hamsters were housed in individually ventilated microisolator cages (4 per cage) with sterile, absorbent beddings changed twice weekly. The animals were fed and watered *ad libitum* throughout the course of the experiment. Following *Leptospira* challenge, hamsters were weighed and monitored daily for endpoint criteria as previously described [8, 55] including loss of appetite, gait or difficulty in breathing, prostration, ruffled fur, and weight loss of ≥ 10% of maximum weight. Hamsters that exhibited these manifestations were euthanized by isoflurane narcosis followed by thoracotomy.

### Plasmid construction

The plasmid construct described in Coutinho *et al*. [18] was used to express LigA Ig-like domains 7’-13 (LIC10465; amino acids 631–1224). The *ligB* (*lic10464*) gene region encoding the amino acids after signal peptide followed by the first seven Ig-like domains (amino acids 19–672) was amplified from *L*. *interrogans* sv. Copenhageni str. Fiocruz L1-130 using primer pair LigB0-7For and LigB0-7Rev (Table 1). The DNA amplicons, which included *Nde*I and *Xho*I restriction sites were ligated into pET-20b(+) (Novagen), provided a carboxy-terminal 6xHis tag. Plasmid constructs were verified by restriction digest and nucleotide sequencing (Table 1). Both LigA7’-13 and LigB0-7 plasmids were transformed into *E*. *coli* expression strain BLR (DE3) pLysS (Novagen). A plasmid containing a shorter gene fragment, *ligA11-13* (encoding amino acids 943–1224, [18]) was also prepared as described earlier using primers LigA11-13For and LigA11-13Rev (Table 1).

**Table 1:** List of oligonucleotides used in this study.

Oligonucleotides (5’ → 3’)		Reference
*Primers used for cloning Lig proteins*		
LigA7’-13 For	AAC ATA TCT CAT ATG CTT ACC GTT TCC AAC ACA AAC GCC AA	[18]
LigA7’-13 Rev	TTC CTC GAG TGG CTC CGT TTT AAT AGA GGC TAA T	[18]
LigB0-7 For	AAA GTC ATA TGT CTT GGC CAC TTT TAA	This study
LigB0-7 Rev	GAA ACT CGA GTG CCG GAG TTA CCT TTA AAA TT	This study
LigA11-13 For	CAT CAA TGA CAT ATG AGA ATA GCT TCA ATC GAA GTA ACA CC	[18]
LigA11-13 Rev	TTC CTC GAG TGG CTC CGT TTT AAT AGA GGC TAA T	[18]
*Primers used for sequencing*		This study
LigA oF2	CCG GTA TCT TCA CCG ACC AC	
LigA oR3	GGA TAG AGG TCA GAA CCG CC	
LigA oF4	CCC GAC CTC TTC TCA CAA AGC	
LigA oR5	CGT TCG GGT CAG AAG AAG ACC	
LigA oF6	TCA AAT CTA ACC GTG CGT GG	
LigB oR1	CGA TGG CTG AGA ATT GAC GA	
LigB oF2	CAC TGT TTC TGC TTC TAG CGA G	
LigB oR3	GAA GTG CGA GTT GGA GAG AC	
LigB oR4	GCT TAC GGA GCA GCT ACA GG	
*qPCR amplification*		[56]
LipL32 45F	AAG CAT TAC CGC TTG TGG TG	
LipL32 268F	GAA CTC CCA TTT CAG CGA TT	
LipL32 189P	FAM AA AGC CAG GAC AAG CGC CG BHQ1	

### Recombinant protein expression and purification

Two-hundred fifty milliliters of 2x YT supplemented with 0.2% dextrose and 100 μg/ml ampicillin was inoculated with a 1:50 dilution of *E*. *coli* containing pET-20b(+) + LigA7’-13 or LigB0-7 overnight culture. The *E*. *coli* cultures were grown at 37^°^C until the optical density at 600 nm (OD_600_) reached 0.5–0.6, and isopropyl-β-D-thiogalactopyranoside (IPTG) (Apex Biotechnology Products) was added at a final concentration of 0.5–1 mM to induce protein expression. The temperature was shifted to 30^°^C, and cultures were incubated for 2 h with agitation at 225 rpm. Cells were harvested by centrifugation at 5,000 *x g* at 4^°^C for 10 min. The bacterial pellet was washed with phosphate buffered saline (PBS) pH 7.2 (Gibco, Grand Island, NY) followed by centrifugation at 5,000 *x g* at 4^°^C for 10 min.

The harvested cells were lysed using BugBuster Protein Extraction Reagent (EMD Millipore, Billerica, MA) in the presence of 0.25 mM phenylmethanesulfonyl fluoride (PMSF) and 10 U/ml DNase I (Thermo Scientific, Rockford, IL), and incubated at room temperature (RT) for 20 min while shaking. The lysate was subjected to centrifugation at 15,000 *x g* for 20 min at 4^°^C. The supernatant was collected, and soluble histidine-tagged proteins were purified by affinity chromatography using Ni-NTA agarose per the manufacturer’s instructions (Qiagen, Valencia, CA) at 4^°^C. After collecting the flow-through, the column was washed 10 times with 20 mM imidazole in 50 mM NaH_2_PO_4_ and 500 mM NaCl (pH 8.0) to remove unbound proteins. His-tagged proteins were eluted from the column with 500 mM imidazole in 50 mM NaH_2_PO_4_ and 500 mM NaCl (pH 8.0). The eluted fractions were analyzed by SDS-PAGE, pooled, and dialyzed extensively in buffer containing 50 mM NaH_2_PO_4_ and 50 mM NaCl (pH 8.0) to remove imidazole. The protein concentration was determined by bicinchoninic acid (BCA) analysis (Pierce, Rockford, IL). When necessary, proteins were concentrated in Amicon centrifugal filter units (EMD Millipore) with a molecular weight cut-off of 10 kDa. The purified recombinant proteins were used immediately for immunization or stored at -80^°^C for subsequent ELISA and western blot analysis.

### Hamster immunization

Recombinant LigA7’-13 and LigB0-7 proteins were expressed and purified prior to every immunization and were delivered as soluble proteins. Animals were immunized as previously described [18]. Groups of five to eight female hamsters, 3–4 weeks of age, were immunized subcutaneously with 100 μg recombinant LigA7’-13, LigB0-7, or 50:50 mixture of LigA7’-13 and LigB0-7, in a total volume of 200 μl with Freund’s complete adjuvant (Sigma-Aldrich) in a 1:1 ratio. The hamsters were boosted at days 14 and 28 with the same amount of protein with Freund’s incomplete adjuvant (Sigma-Aldrich) in a 1:1 ratio. Control groups included animals that were immunized with PBS alone or PBS with adjuvant. Blood was collected *via* retro-orbital route 3 days before the first immunization (pre-bleed) and weekly thereafter (days 4, 11, 18, 25, and 32) until animals were challenged with *L*. *interrogans* at day 35 post-immunization. Serum was prepared from collected blood by centrifugation at 21,100 *x g* for 15 min, and was stored at -80^°^C until use.

### *Leptospira* challenge and sample collection

One week after the last immunization, hamsters were challenged intraperitoneally with 1 ml of 1 x 10^4^ *L*. *interrogans* sv. Copenhageni st. Fiocruz L1-130 (≥ 500-fold over ED_50_) [8]. The animals were weighed daily and monitored for endpoint criteria [8, 55] as described earlier. Hamsters that survived infection were euthanized 28 days post-challenge. At the time of sacrifice, one kidney was collected for culture in EMJH, while the other kidney and liver were used for quantification of *L*. *interrogans* by quantitative PCR (qPCR). Serum prepared from blood collected by post-mortem cardiac puncture were used for serologic analyses and microscopic agglutination test (MAT).

Collected kidney tissues were pulverized by passing them through a 3 ml syringe and inoculated into a tube containing semisolid EMJH supplemented with 100 μg/ml 5-fluorouracil. A 1:100 dilution was passaged to a new semisolid medium and the tubes were incubated at 30^°^C. The cultures were checked weekly for the presence of *L*. *interrogans* by dark field microscopy for up to 28 days, before being designated negative. Positive cultures generally show up between 7–14 days.

### Quantitative PCR

Hamster kidney and liver tissues were prepared for DNA extraction by dicing 50–80 mg of tissue and suspending in 500 μl PBS. Following tissue homogenization for 1 min at 5 movements per second (OMNI Bead Ruptor 24, Omni International, Kennesaw, GA), total genomic DNA was extracted from equivalents of 25 mg tissue using the DNeasy Blood and Tissue Kit (Qiagen) following the manufacturer’s instructions but eluted with 100 μl of elution buffer. DNA was prepared for the standard curve by growing serovar Copenhageni in EMJH culture medium to a density of 4 x 10^5^ spirochetes/ml. DNA was extracted from 5 ml culture using the same kit. The concentration of eluted DNA was determined using a spectrophotometer at OD_260_ (GE NanoVue Spectrophotometer). All DNA samples were kept at -80^°^C until use.

*Leptospira* DNA (1 x 10^0^ to 1 x 10^7^ genomic equivalents (GEq) /5 μl) was prepared as previously described [57]. *L*. *interrogans* genome was quantified by qPCR using Bio-Rad iTaq Universal Probes supermix (Bio-Rad, Hercules, CA) supplemented with 250 nM *lipL32* forward (45F) and reverse (268R) primers, and 150 nM *lipL32* probe (189P) [56] (Table 1). Five microliters of standard or sample DNA was added to 15 μl PCR mix. The amplification protocol is as follows: 3 min at 95^°^C, followed by 40 cycles at 95^°^C for 15 s and 60^°^C for 1 min. All standards and samples were run in duplicate. A standard curve was generated using Bio-Rad iQCycler5 software, and the number of GEq was extrapolated from the threshold cycle (C_*T*_) values. A negative result was assigned where no amplification occurred or if the C_*T*_ value was greater than 36 [56]. Data are presented as number of *L*. *interrogans* GEq per gram of tissue. Uninfected hamster liver and kidney tissues were used as negative controls to determine the limit of detection (LOD) of the assay.

### Microscopic agglutination test (MAT)

Kidney culture-negative samples were subjected to MAT as described by Goris and Hartskeerl [58]. Sera were heat-inactivated at 56^°^C for 30 min. Serial two-fold dilutions of serum (1:10 to 1:10,240) were prepared in PBS pH 7.2, and incubated at 30^°^C for 30 min with equal volumes of *L*. *interrogans* sv. Copenhageni st. Fiocruz L1-130 (optical density at 420 nm (OD_420_) = 0.2) in a 96-well plate. Cultures were examined under dark-field microscopy for bacterial agglutination. The titer was determined as the highest dilution > 50% of the bacteria are freely moving compared to control suspension without sera. Sera from uninfected hamsters were used as controls.

### Enzyme-linked immunosorbent assay (ELISA)

Serum antibody responses to recombinant protein immunization were quantified by ELISA as previously described [8, 18] with few modifications. ELISA was performed by coating microtiter plates (Immulon 4HBX, Thermo Scientific) with 1 μg of recombinant protein per well (100 μl diluted in PBS, pH 7.2) overnight at 4^°^C. The wells were washed three times with 200 μl PBS, blocked with 200 μl protein-free blocking buffer in PBS (PFB) (Pierce) for 1 h at RT, then incubated with 100 μl 1:6,400 dilutions of hamster sera in PFB for 1 h at 37^°^C. The plates were washed three times with PBS and incubated with 100 μl horse radish peroxidase (HRP)-conjugated goat anti-Syrian hamster IgG (1:5,000 in PFB) (Jackson ImmunoResearch, West Grove, PA) for 30 min at RT. After 3 washes with PBS, plates were developed with 100 μl 3,3′,5,5′-tetramethylbenzidine (1-step Turbo TMB HRP substrate, Thermo Scientific) for 15 min at RT in the dark. The absorbance at 655 nm (OD_655_) was determined using an automated plate reader (Bio-Rad 550 Microplate Reader, Bio-Rad). The IgG response was expressed as relative α-Lig antibody level, determined by subtracting the absorbance of the pre-immune bleed (baseline or background read) from the immune sera collected at different time points. All experiments were done in triplicate.

### Immunoblot analysis

Immunoblot analysis were performed to determine if sera from immunized animals recognize purified His-tagged or native Lig proteins, and to determine the antigenicity of the subunit vaccines. *L*. *interrogans* lysate was prepared by growing cultures to mid-log phase, followed by induction with 120 mM NaCl for 4 h at 30^°^C, 150 rpm. Bacteria were harvested by centrifugation at 15,000 *x g* for 15 min, washed with PBS, and centrifuged again to collect the pellet. The bacterial pellet was resuspended in SDS protein sample buffer supplemented with dithiothreitol (DTT) (Thermo Scientific), boiled for 5 min, and kept at -20^°^C until use.

Fifteen microliters of *L*. *interrogans* whole cell lysate or 100–200 ng/well purified recombinant Lig proteins or control leptospiral lipoprotein LipL32 were separated by 10% or 4–12% SDS-PAGE (NuPage Bis-Tris protein gels, Life Technologies, Carlsbad, CA) and transferred to polyvinylidene difluoride (PVDF) membrane (Immobilon, EMD Millipore) for western blotting. The membranes were blocked with 5% skim milk in PBS-Tween 20 (Thermo Scientific) overnight at 4^°^C. The membranes were cut into strips and incubated in pooled or individual hamster sera (pre-bleed or last bleed) from immunized animals or control rabbit α-Lig antiserum (at dilutions 1:2,000–1:10,000 in 5% milk in PBS-Tween 20) for 1 h at RT over a rocker. The Lig antiserum, which reacts with LigA and LigB, was raised against amino acids 342–1224 of *L*. *kirschneri* LigA and has been described previously [28]. As loading control for *Leptospira* lysate, membranes were probed with rabbit α-LipL41 (1:10,000 dilution). As loading controls for recombinant LipL32, membranes were probed with rabbit α-Lip32 (1:5,000 dilution) and mouse α-6xHis epitope (1:1,000 dilution, Thermo Scientific). To test the antigenicity of subunit vaccines, membrane strips were incubated with 1:250 dilution of the following: pooled sera from hamsters infected intraperitoneally with Copenhageni serovar, and corresponding pre-bleeds as control; pooled sera obtained from convalescent patients diagnosed with leptospirosis (provided by Dr. Albert Ko, Yale University, New Haven, CT), and normal human serum (Millipore) as control.

After incubation with primary antisera, the membranes were washed three times with PBS-Tween 20 and were probed with horseradish peroxidase (HRP)-conjugated α-hamster (1:5,000 dilution, Jackson ImmunoResearch), α-rabbit (1:5,000–1:10,000 dilution, GE Healthcare), α-mouse (1:5,000 dilution, GE Healthcare), or α-human (1:20,000 dilution, Sigma-Aldrich) antibodies for 40 min at RT. Membranes were washed and the antigen-antibody binding was detected by using enhanced chemiluminescence (ECL) system. Blots were incubated in ECL reagent (Pierce) for 1 min, exposed to a film (Amersham HyperFilm ECL), and developed (Konica SRX-101A).

### Statistical tests

The differences in survival and mortality rates among experimental groups were determined using the Log-rank (Mantel-Cox) test and Fisher’s exact test, respectively. Kruskal-Wallis test was used to determine whether there is a significant difference in the number of bacteria in kidney or liver among the survivors from different immunization groups while one-way analysis of variance (ANOVA) was used to compare immune responses among animals in the same treatment group. For all tests, statistical significance was set to *P* ≤ 0.05. All analyses were performed using Graph Prism 5 (GraphPad Software, Inc., La Jolla, CA).

## Results

### Preparation of purified recombinant Lig proteins

**Fig 1:**
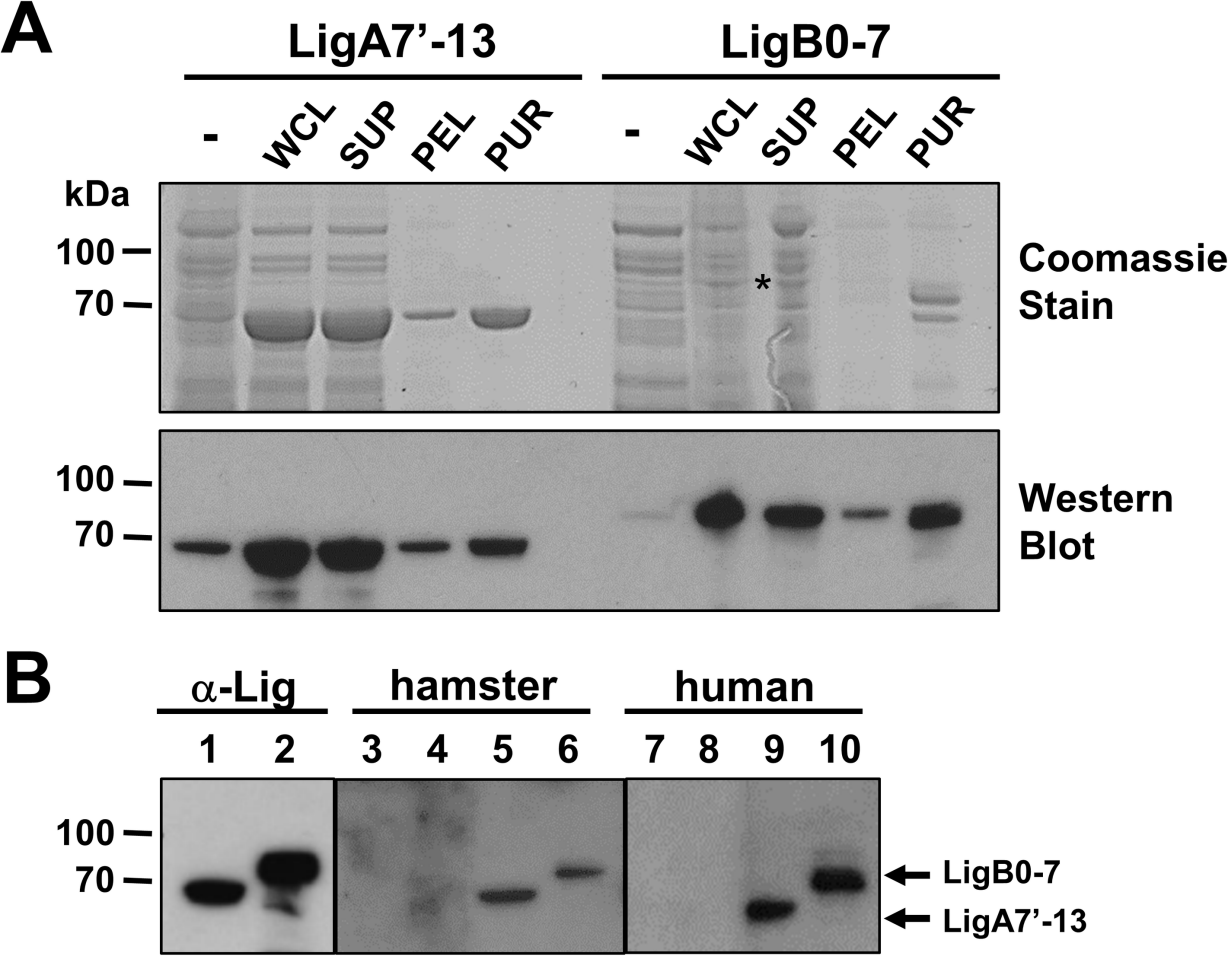
Expression, purification, and antigenicity of recombinant His-tagged Lig proteins. (A) *E*. *coli* BLR (DE3) pLysS transformed with pET-20b(+) containing *ligA7’-13* or *ligB0-7* were grown to an OD_600_ = 0.5–0.6 prior induction with 0.5–1 mM IPTG for 2 h at 30^°^C. Bacterial cells were harvested by centrifugation, followed by lysis in the presence of protease inhibitors. Supernatant was separated from pellet fraction by centrifugation at 16,000 *x g* at 4^°^C. His-tagged Lig proteins were purified from the supernatant fraction by affinity chromatography using Ni-NTA resin. Fifteen microliters of whole cell lysates from uninduced (-) and induced cultures (WCL), supernatant (SUP) and pellet (PEL) fractions, and 2 μg purified protein (PUR) were separated by 4–12% SDS-PAGE followed by Coomassie staining. A replicate gel was also run loaded with 5 μl cell fractions and 200 ng purified proteins for immunoblot analysis. The proteins were transferred to a PVDF membrane and the blot was probed with rabbit α-Lig antiserum (dilution 1:10,000). The expressed His-tagged LigA7’-13 and LigB0-7 were found predominantly in the supernatant. Asterisk shows LigB0-7, produced at lower levels compared to LigA7’-13. (B) Two hundred nanograms of LigA7’-13 (lanes 1, 3, 5, 7, 9) or LigB0-7 (lanes 2, 4, 6, 8, 10) were run in 4–12% SDS-PAGE gel and transferred to PVDF. Membranes were probed with 1:10,000 control α-Lig antiserum (lanes 1, 2), or 1:250 of the following: pooled sera from hamsters before (lanes 3, 4) and after (lanes 5, 6) intraperitoneal challenge with *L*. *interrogans*, normal human serum (lanes 7, 8), and pooled sera from leptospirosis patients in convalescent stage (lanes 9, 10). Both Lig proteins were recognized by sera from *Leptospira-*infected hamsters and humans.

To test the protective properties of Lig proteins, recombinant LigA7’-13 and LigB0-7 proteins were prepared. The segment of *ligA* encoding LigA7’-13 was cloned and expressed as previously described [18]. The LigB0-7 construct starts after the signal sequence and spans the first through seventh Ig-like repeat domains of the LigB protein. Comparison of the amino acid sequences of the LigA7’-13 and LigB0-7 using Basic Local Alignment Search Tool (BLAST) shows that the sequence identity between the two proteins is 66%. Both LigA7’-13 and LigB0-7 were expressed as soluble proteins, although LigA7’-13 expression in *E*. *coli* was more robust compared to LigB0-7 (Fig 1). The expressed proteins contain 6xHis tag at the C-terminal region and were purified under non-denaturing conditions by affinity chromatography using a nickel-charged resin. Both proteins were dialyzed extensively to remove imidazole in the solution. Purified, recombinant LigA7’-13 was stable in solution, however, precipitation was observed in purified LigB0-7 when kept for prolonged periods at 4^°^C. We evaluated the antigenicity of the purified Lig proteins by immunoblot analysis and demonstrated that both LigA7’-13 and LigB0-7 were recognized by sera from hamsters infected intraperitoneally with *L*. *interrogans*, and by sera of leptospirosis patients from Brazil in convalescent phase (Fig 1).

### Humoral response of Lig immunized hamsters

**Fig 2:**
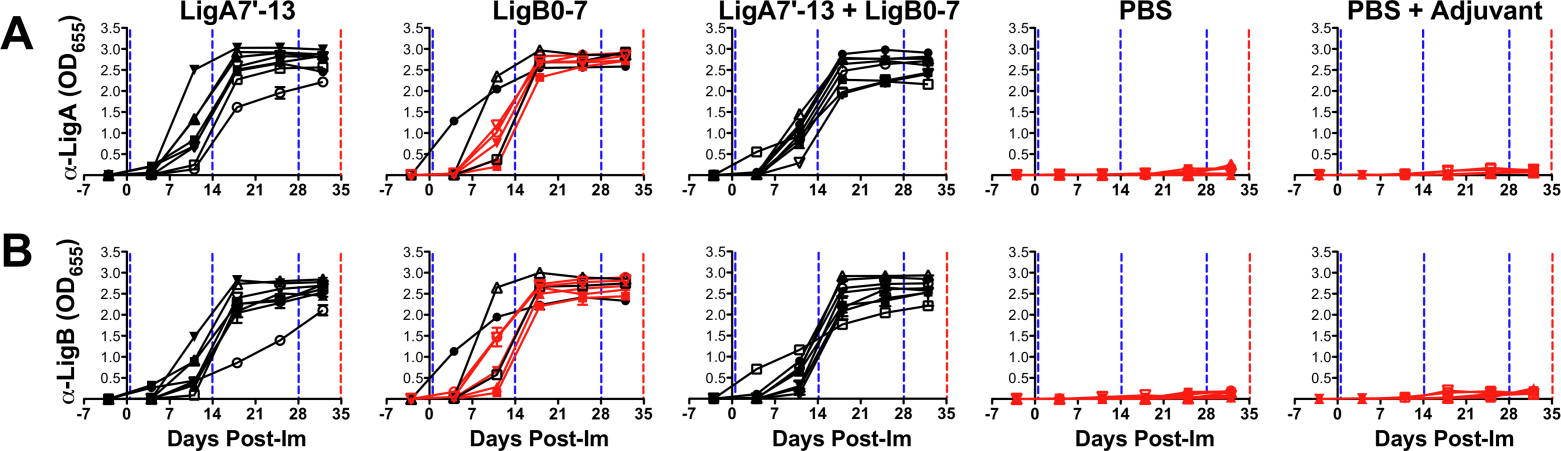
IgG response to immunization with purified recombinant Lig proteins. Serum samples were collected from hamsters weekly during the immunization protocol. Anti-LigA7’-13 (A) or anti-LigB0-7 (B) antibody levels were measured in triplicate by ELISA. Each data line represents the IgG response of an individual animal over time; in *black* are animals that survived the challenge while in *red* are animals that met the endpoint criteria. Vertical dotted lines indicate immunization days (blue) or challenge with *L*. *interrogans* (red). Each data point represents the mean IgG level read at OD_655_ minus pre-bleed read. Error bars indicate standard deviation. There was no significant difference in the IgG response among hamsters that received the same vaccine treatment at all time points (One-way ANOVA, *P* = 0.9).

**Fig 3:**
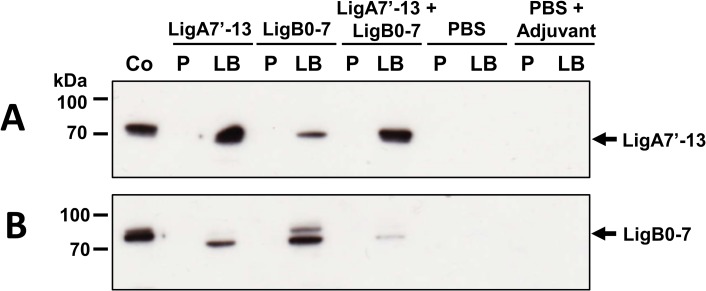
Recognition of purified, recombinant LigA7’-13 and LigB0-7 proteins by pooled sera from immunized hamsters. One to two hundred nanograms per well of purified His-tagged LigA7’-13 (A) or LigB0-7 (B) were run in 4–12% SDS-PAGE then transferred to PVDF membrane for western blot analysis. The membrane was cut to strips and probed with 1:5,000 pooled sera collected before immunization (pre-bleed, P) and at day 32 (last bleed, LB) from immunized and control groups. Another membrane strip was incubated with 1:5,000 rabbit α-Lig as positive control (Co). Sera collected from LigA7’-13-vaccinated animals cross reacted with recombinant LigB0-7 and vice versa.

To assess whether animals immunized with Lig proteins developed an IgG antibody response, sera were collected weekly during the immunization protocol and examined by ELISA using LigA7’-13 and LigB0-7 antigens. As presented in Fig 2, there was no detectable IgG against either purified protein in serum from hamsters in both control groups, PBS and PBS with adjuvant. Immunization with LigA7’-13 or LigB0-7 produced IgG that recognizes the homologous recombinant protein fragment. Sera from LigA7’-13 + LigB0-7 immunized hamsters react with both Lig protein fragments. The antibody responses observed were directed towards the immunized Lig protein, and not to the epitope tag, as sera from both control and immunized groups did not recognize a non-relevant 6xHis-tagged leptospiral lipoprotein LipL32 (S1 Fig). However, cross-reactivity was observed as sera from LigA7’-13 vaccinated animals reacted against recombinant LigB0-7 immobilized on ELISA plates and vice versa. Cross-reactions were confirmed by immunoblot analysis, probing recombinant Lig proteins immobilized on PVDF membrane with pooled sera from immunized animals (Fig 3).

The IgG response of individual animals that received the same vaccine treatment were comparable at all time points (one-way ANOVA, *P =* 0.9) (Fig 2). A substantial increase in α-LigA7’-13 and α-LigB0-7 antibody titers was observed at day 18, after the first immunization boost and IgG antibody levels were maintained until the leptospiral challenge at day 35 (Fig 2 and S2 Fig). There was no significant difference in the α-LigA7’-13 IgG levels among treatment groups LigA7’-13, LigB0-7, or combination, at all time points (S2 Fig). The α-LigB0-7 IgG level in LigB0-7 immunized hamsters was significantly higher one week after the first immunization (day 11 bleed) compared to animals of the other vaccine groups. However, antibody titers were comparable to other vaccine groups at later time points (S2 Fig). These results suggest that both LigA7’-13 and LigB0-7 immunizations elicit humoral immune response in hamsters.

### Protective properties of Lig vaccines against *L*. *interrogans* challenge

Recombinant Lig antigens were evaluated for their protective properties using a hamster model of acute leptospirosis. Animals were challenged intraperitoneally one week after the last immunization with 1x10^4^ *L*. *interrogans* sv. Copenhageni str. Fiocruz L1-130 (~500-fold the ED_50_ [8]). The challenge strain used was at a low passage (2) and expresses LigA and LigB when incubated at physiological osmolarity (S3 Fig).

**Fig 4:**
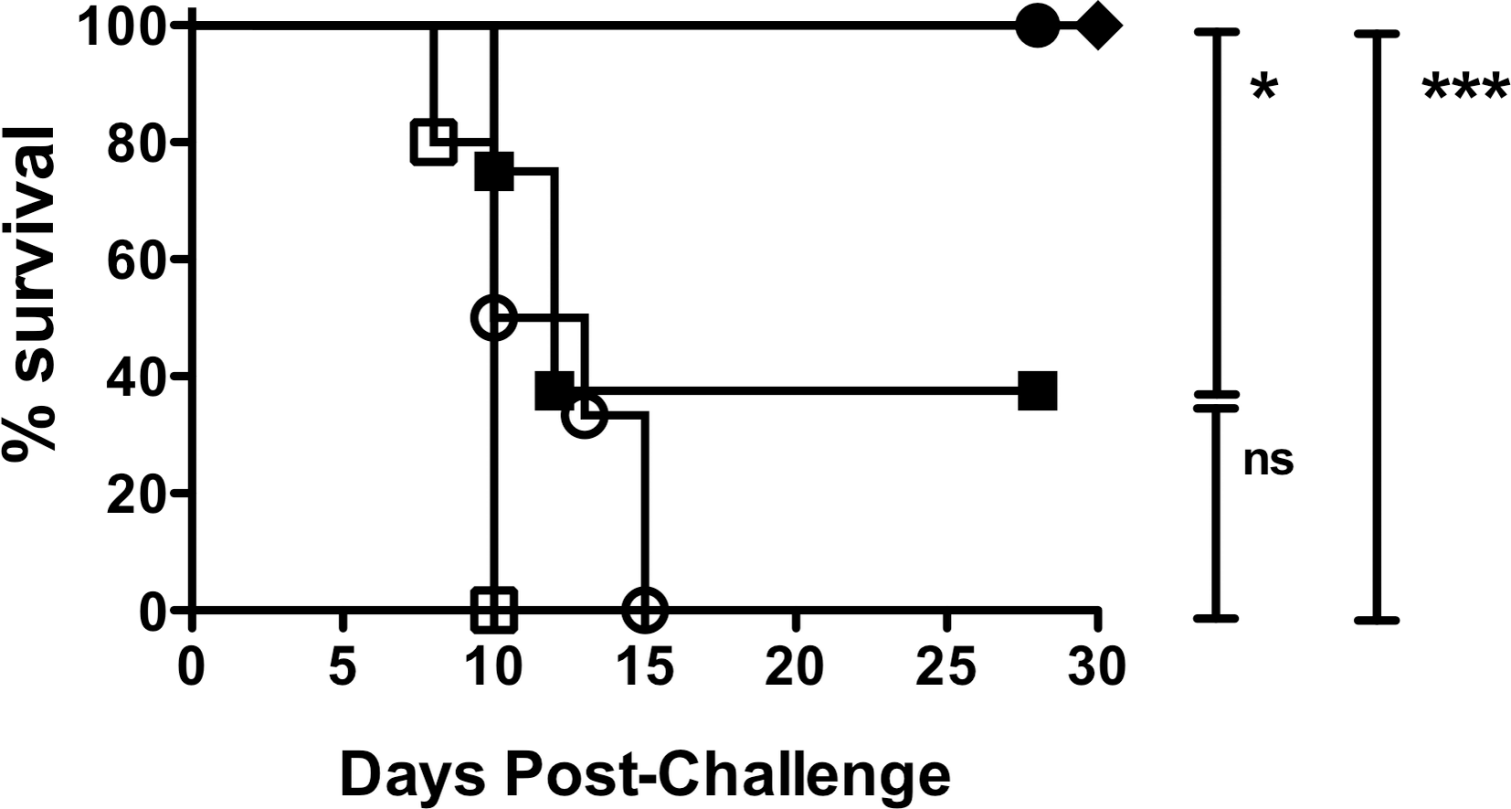
Survival of hamsters immunized with purified recombinant Lig proteins after lethal challenge. Groups of 5–8 female hamsters were immunized subcutaneously three times with 100 μg LigA7’-13 (●), LigB0-7 (■), LigA7’-13 + LigB0-7 (◆), or controls PBS (○) and PBS with adjuvant (□). Animals were challenged intraperitoneally with 1 x 10^4^ *L*. *interrogans* at day 0 and post-challenge survival was followed until day 28. Asterisks indicate significant difference between mortality rates compared (Fisher’s exact test, *P < 0.05, ***P < 0.001, ns not significant).

Hamsters were observed and their weights monitored daily after challenge, as weight loss has been found to be an early objective sign of clinical leptospirosis [18]. PBS or PBS with adjuvant sham immunized animals started to lose weight as early as day 7 post-challenge and by day 15, all hamsters in both groups met the endpoint criteria (Fig 4 and Table 2). Two hamsters from the PBS with adjuvant group succumbed to *Leptospira* challenge before showing signs of the disease or reaching the 10% weight loss designated as our endpoint. In contrast, all LigA7’-13 and LigA7’-13 + LigB0-7 immunized hamsters survived the intraperitoneal bacterial challenge. LigB0-7 vaccinated animals exhibited 37.5% survival; five of the eight LigB-immunized animals showed signs of leptospirosis 8–12 days post-challenge. The difference between the survival rates of LigB0-7 vaccinated animals and control animals (PBS and PBS with adjuvant groups) was not statistically different (Fisher’s exact test, *P* = 0.2088 and *P* = 0.2308, respectively) (Fig 4). In addition, no difference was observed in IgG response among all LigB0-7 immunized animals whether they survived infection or met endpoint (Fig 2). Two of the LigB0-7 immunized survivors exhibited 4.1% and 4.2% weight loss starting at day 7 or 8, but did not meet the endpoint criteria set for weight loss at ≥ 10%, and were able to recover their weights by day 11. We did not observe any significant weight loss among LigA7’-13 or LigA7’-13 + LigB0-7 immunized hamsters following bacterial challenge.

**Table 2:** Protection conferred by immunization with recombinant Lig proteins against intraperitoneal *L*. *interrogans* challenge.

Vaccine groups	No. animals that survived challenge/total animals (%)	No. animals that met endpoint criteria/total animals (endpoint days)	No. animals that are culture positive/total animals
LigA7’-13	8/8 (100%)	0/8	8/8
LigB0-7	3/8 (37.5%)	5/8 (10,10,12,12,12)	7/8*
LigA7’-13 + LigB0-7	8/8 (100%)	0/8	8/8
PBS	0/6 (0%)	6/6 (10,10,10, 13,15,15)	6/6
PBS + Adjuvant	0/5 (0%)	5/5 (8,10,10, 10,10)	5/5

*One of the hamsters that survived the intraperitoneal challenge; qPCR was below LOD; negative MAT result.

### Sterilizing immunity conferred by Lig protein immunization

Pathogenic *Leptospira* spp. disseminate in the blood and reach target organs including the kidney [1, 2]. To determine if Lig protein immunization provides protection against renal colonization, kidneys collected from hamsters were cultured in semisolid EMJH supplemented with 5-fluorouracil, and checked weekly under dark field microscope for the presence of motile *Leptospira*. As presented in Table 2, all kidney tissue cultures collected from sham-immunized animals were positive. Similar to previous studies [8, 18, 22, 26], kidneys of LigA7’-13 immunized survivors were colonized with leptospires. The addition of LigB0-7 to LigA7’-13 vaccine did not assist in eradication of the bacteria from the kidneys; all LigA7’-13 + LigB0-7 vaccinated animals had positive kidney cultures. One of the hamsters in the LigB0-7-immunized group was culture-negative, and found to be MAT-negative when tested at serum dilutions 1:10 to 1:10,240.

**Fig 5:**
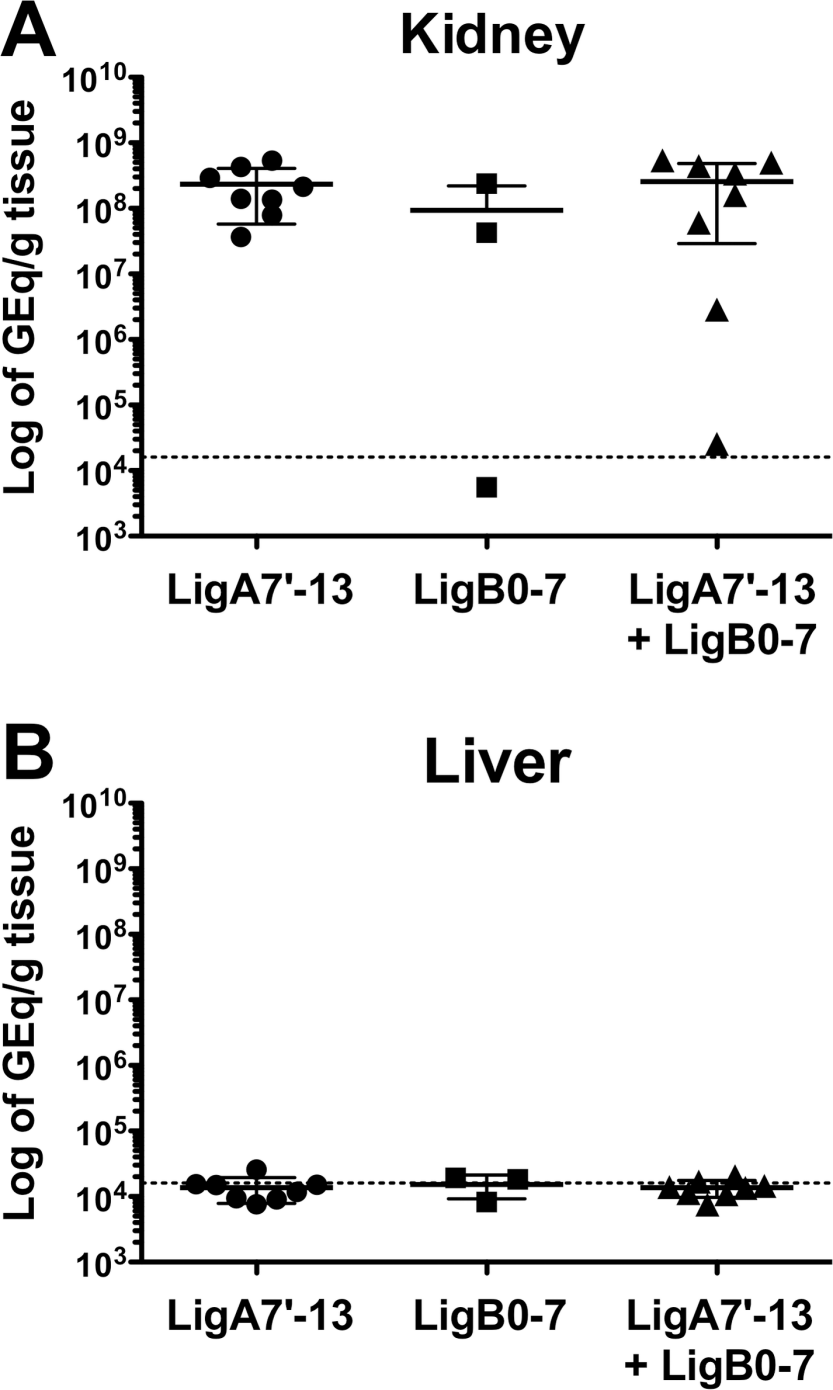
Bacterial load in kidney and liver of hamsters that survived the *L*. *interrogans* challenge. Total genomic DNA was extracted from kidney (A) and liver (B), and analyzed by qPCR performed in duplicates with *lipL32* primers and probes (Table 1) to quantify leptospiral tissue load. Bacterial burden was expressed as genomic equivalents (GEq) per gram of tissue. Black lines show mean bacterial load with error bars representing standard deviation, while dotted lines indicate limit of detection. There was no difference in the bacterial burden in kidneys among the different vaccine groups (Kruskal-Wallis test, *P* = 0.4050).

*Leptospira* in both liver and kidney were quantified by qPCR to accurately assess the difference in bacterial load among survivors of the three vaccine groups. As shown in Fig 5, we did not detect *L*. *interrogans* in the liver of hamsters 28 days post-challenge. On the other hand, we observed high bacterial burdens in kidneys with up to 10^8^ GEq per gram of tissue among survivors. However, there was no significant difference in the kidney burdens among hamsters from the three different treatment groups (Kruskal-Wallis test, *P* = 0.4050). The bacterial kidney load in one of the LigB0-7 immunized hamster was below the set limit of detection; this hamster was also kidney culture-negative and MAT-negative. These results suggest that LigB0-7 does not protect hamsters from *L*. *interrogans* challenge, nor does the addition of LigB0-7 to LigA7’-13 lower bacterial burden in kidneys.

### Recognition of native Lig proteins by sera from immunized animals

To determine if IgG produced from Lig protein immunization of hamsters recognize native proteins, we incubated *L*. *interrogans* in the presence of 120 mM NaCl for 4 h at 30^°^C. After osmotic induction, the bacteria were harvested by centrifugation, washed with PBS, and resuspended in sample buffer. *Leptospira* lysates were immobilized on PVDF membranes and probed with pooled or individual sera from control and treatment groups collected prior to leptospiral challenge.

**Fig 6:**
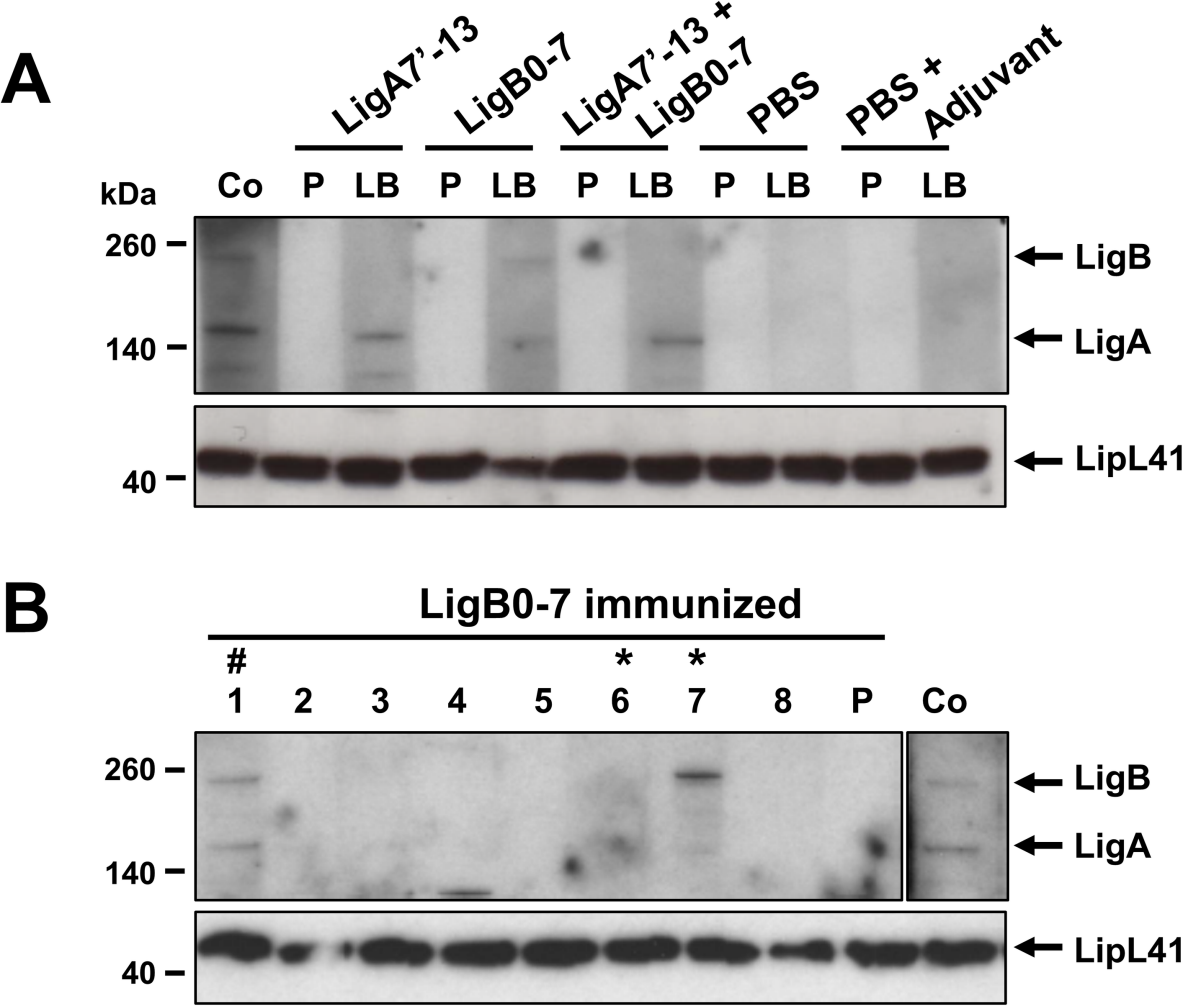
Recognition of native LigA and LigB in *Leptospira* lysate by sera from immunized hamsters. A *L*. *interrogans* culture at an OD_420_ = 0.29 was incubated in EMJH supplemented with 120 mM NaCl for 4 h at 30^°^C. Bacteria was harvested by centrifugation, washed with PBS, resuspended in sample buffer, and boiled for 5 min. Fifteen microliters of bacterial lysate was separated in 4–12% SDS-PAGE, then transferred to a PVDF membrane for western blot analysis. (A) The membrane was cut to strips and probed with pooled sera (1:2,000 dilution) collected before immunization (pre-bleed, PB), and at day 32 (last bleed, LB) from immunized and control hamsters. (B) The membrane strips were probed with sera (1:2,000 dilution) from individual LigB0-7 vaccinated hamsters (1–8). The prominent band recognized by serum from hamster 7 migrates slower than LigB. The membranes were also probed with rabbit α-Lig (Co, 1:2,000 dilution) and rabbit α-LipL41 (1:10,000 dilution) as controls. Asterisks (*) represent hamsters that survived the challenge, while pound sign (#) designates animal that survived and was culture- and MAT-negative.

Fig 6A shows that pooled sera from LigA7’-13 immunized animals bind to native LigA. On the other hand, pooled sera from LigB0-7 vaccinated hamsters recognize both native LigA and LigB proteins, although LigA signal was weak. The cross-reactivity with native LigA is possibly due to high sequence similarity (94%) between LigB0-7 and the corresponding LigA0-7 fragment (amino acids 19–672). However, immunization with both proteins generated antibodies that recognize LigA only. Since these hamsters were immunized with half the amount of each protein, we speculate the LigB0-7 dosage used was too low to elicit a robust production of antibodies that bind native LigB protein. *Leptospira* proteins were not detected by pre-bleed sera nor by sera collected from mock-immunization groups.

We examined individual sera from LigB0-7 immunized hamsters to determine if there is a difference in the recognition of native Lig proteins among animals that survived or met the endpoint criteria. None of the sera from hamsters that succumbed to the bacterial challenge recognized native LigA or LigB (Fig 6B). Serum from only one survivor (hamster 1) recognized both LigA and LigB; serum is from the animal from which we were not able to recover *Leptospira* from the kidney. Sera from the two other survivors (hamsters 6 and 7) did not recognize LigB. The prominent band observed when probed with serum from hamster 7 had a higher molecular weight compared to LigB. However, the serum from this animal recognized the native LigA. These results indicate that when leptospiral lysate was probed by pooled sera from LigB0-7 immunized animals (Fig 6A), the higher molecular weight band observed is predominantly from a non-relevant protein recognized by hamster 7 sera and in part contributed by hamster 1 sera binding to native LigB. We repeated the assay using a higher concentration of sera (1:500 dilution) in an effort to enhance some possibly weak signals of the other sera samples but observed the same results as shown in Fig 6B.

### Recognition of domains 11–13 of LigA is by sera from immunized hamsters

**Fig 7:**
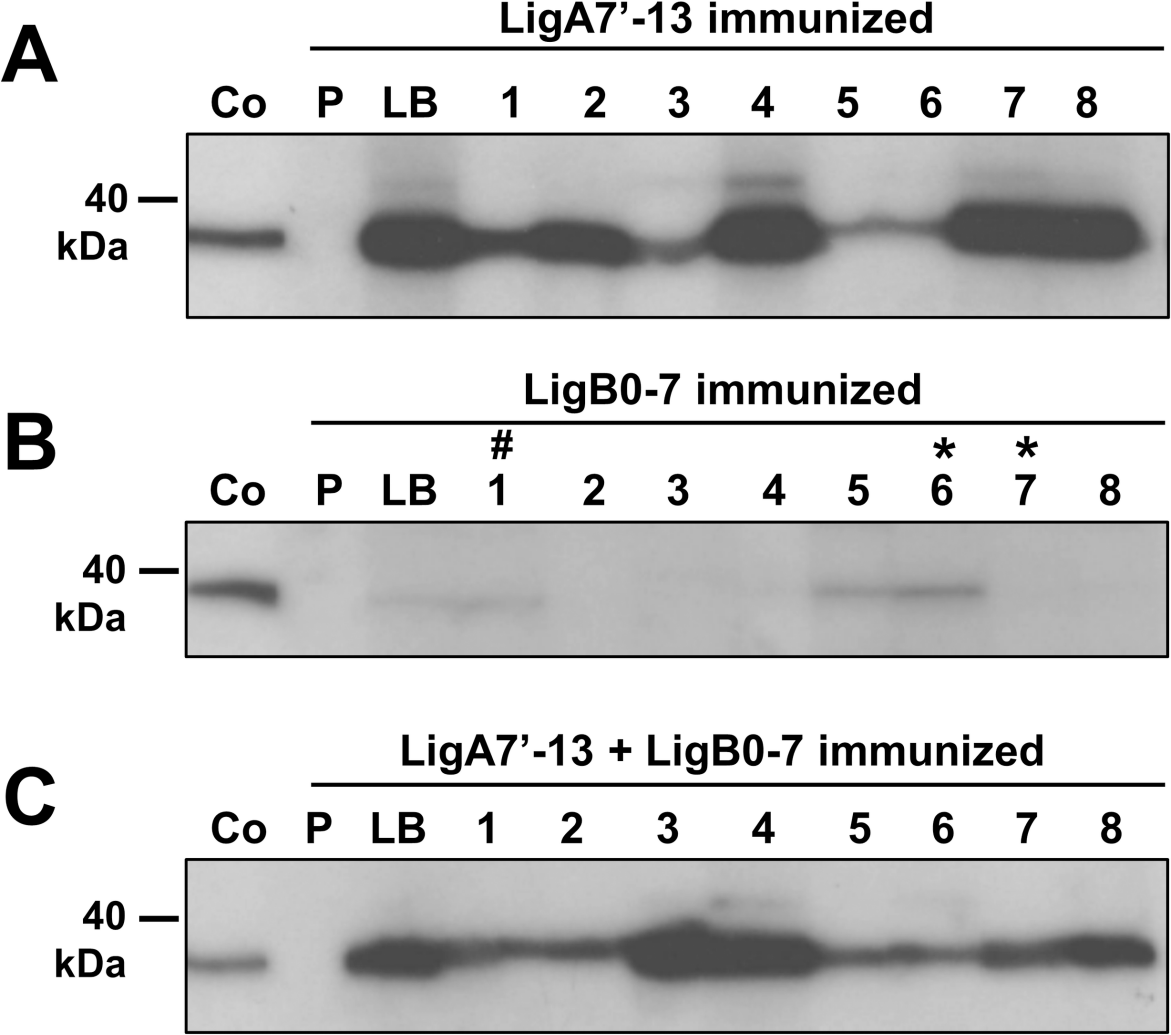
Recognition of purified, recombinant LigA11-13 proteins by sera from immunized hamsters. One hundred nanograms per well of purified His-tagged LigA11-13 were run in 4–12% SDS-PAGE then transferred to a PVDF membrane for western blot analysis. The membrane was cut to strips and probed with 1:3,000 pooled sera collected before immunization (pre-bleed, P) and at day 32 (last bleed, LB), and sera from individual hamsters immunized with LigA7’-13 (A), LigB0-7 (B), and LigA7’-13 + LigB0-7 (C). Another membrane strip was incubated with 1:3,000 rabbit α-Lig as positive control (Co). In Panel B, asterisks (*) represent hamsters that survived the challenge, while pound sign (#) designates animal that survived and was culture- and MAT-negative. All animals immunized with LigA7’-13 alone, or in combination with LigB0-7, survived the challenge.

Both ELISA and immunoblot analysis showed that IgG produced by vaccinating with purified His-tagged LigA7’-13 or LigB0-7 strongly recognizes homologous protein, and cross-reacts with heterologous protein. We prepared a shorter construct of *ligA*, consisting of domains 11–13, which has been described as sufficient for immunoprotection [18]. By immunoblot analysis, we observed that sera from LigA7’-13 or LigA7’-13 + LigB0-7 immunized survivors recognized recombinant LigA11-13 protein (Fig 7). This construct has a 41% sequence identity with LigB0-7 protein (BLAST). Only three sera from eight LigB0-7 immunized animals recognized LigA11-13 (Fig 7). Interestingly, two (hamster 1 and 6) out of the three sera are from hamsters that survived the *L*. *interrogans* challenge.

## Discussion

In an effort to develop a vaccine that confers protection across all pathogenic serovars and protects the kidneys from bacterial colonization, we tested the immunoprotective properties of LigA7’-13 in combination with LigB0-7. We confirmed results of previous studies that the immunization of hamsters with LigA7’-13 protected hamsters from death but not from infection, while animals that received LigB0-7 protein were mostly susceptible to leptospiral infection [18, 26]. We found that addition of LigB0-7 to LigA7’-13 neither conferred sterilizing immunity nor reduced the bacterial burden in kidneys.

Earlier studies suggested that the mechanism of protection against leptospirosis is mediated largely by the humoral immune response [3, 59]. We observed high titers of antibodies recognizing the homologous protein, and a strong cross-reactivity of IgG antibodies against the heterologous recombinant Lig protein due to the high sequence conservation between the two constructs. Although hamsters immunized with LigB0-7 generated antibodies against the protective fragment LigA7’-13, immunization with LigB0-7 did not protect animals from death. We therefore tested sera from immunized animals for the recognition of domains 11–13 of LigA as this was described to be sufficient in protecting hamsters from infection [18]. These domains are found in the unique region of LigA described to attach to the gelatin binding domain of fibronectin [37]. In addition, LigA 11–13 overlaps with the domains that facilitate binding to the complement regulator C4b-binding protein (C4BP) [60]. Sera from two out of the three LigB0-7 immunized animals that survived the challenge cross-reacted with LigA11-13 by immunoblot assay. These findings raise the possibility that LigA domains that mediate attachment to the extracellular matrix and involved in the capture of a complement regulator are sufficient to protect animals from infection and warrant further studies to determine the mechanisms involved in LigA immunoprotection.

Despite a strong humoral response of all LigB0-7 immunized animals, only three of the eight survived *Leptospira* challenge, with sterilizing immunity achieved in one case. A possible explanation for this observation is that the other seven animals failed to generate antibody that reacts against native LigB produced by *L*. *interrogans*. Previous reports have showed LigBrep (corresponding to our LigB0-7 construct) protected kidneys from bacterial colonization when administered as a DNA vaccine [21, 61]. In this context, it would be of interest to determine whether sera from animals immunized with LigBrep DNA recognize the native LigB protein. However in these studies, bacterial burden in kidneys was determined primarily by culture or by imprinting tissue samples on glass plate and probing with anti-*Leptospira* antibodies–methods that may be less sensitive and can generate false negative results. Using qPCR, we concluded that the addition of LigB0-7 to LigA7’-13 failed to lower bacteria burdens in kidneys.

Although LigB0-7 was found predominantly in the soluble fraction and purified under non-denaturing conditions, we noticed protein precipitation during long term storage. The instability of LigB0-7 in solution may have resulted in the loss of native structure and subsequent loss of integrity of epitopes necessary to elicit protective antibodies. In the study conducted by Murray and colleagues [62], out of the 223 *L*. *borgpetersenii* proteins predicted to be surface-exposed, only 12% were expressed and purified in *E*. *coli* as soluble proteins. The remaining proteins were purified under denaturing conditions and none of these antigens were found to be protective in a hamster model of infection. In fact, the disparity in the reported claims of protection conferred by LigB as subunit vaccine may in part be due to the differences in the solubility and/or epitopes of proteins used for immunization [17, 26, 27, 61]. It should be noted that the protection conferred by soluble rLigBcon (corresponding to LigB0-7 construct) immunization described by Yan *et al*. [27] has some caveats, as discussed elsewhere [10]. Their group used a less virulent *Leptospira* strain at a very high challenge dose and were unable to induce lethal infection in all control animals in three independent experimental trials.

A recent study by Conrad *et al*. [63] demonstrated that a subunit vaccine preparation of the conserved region of LigB (amino acids 131–645) conferred sterilizing immunity. Similar to other Lig vaccine studies, the expressed protein was found mostly in the insoluble fraction and purified under denaturing conditions followed by a dialysis step to refold the recombinant protein. The LigB (131–645) construct described lacks the region between the signal peptide and Ig-like domain 1 and the C-terminal segment of the Ig-like domain 7 present in our LigB0-7 (19–762) that potentially rendered our protein predominantly soluble. In our study, the hamsters were immunized subcutaneously with a mixture of LigB0-7 and Freund’s adjuvant. Conrad *et al*. described that intramuscular immunization of LigB (131–645) adsorbed on aluminum hydroxide protected 80–100% hamsters against intraperitoneal challenge of 200 (10x ED_50_) *L*. *interrogans* sv. Copenhageni str. Fiocruz L1-130. Additionally, the kidneys of hamsters that survived bacterial challenge were *Leptospira*-negative as assessed by culture method and/or by qPCR. This is the first evidence of protection both from lethal infection and renal colonization conferred by a Lig subunit vaccine. The findings of their group, along with our results, highlight the importance of immunogen selection, recombinant protein preparation method, route of administration, adjuvant selection, immuogen dosage, and frequency of immunization as determinants in design of potential subunit vaccines.

Studies in cattle generated a mounting interest in exploring the role of cell-mediated responses in immunity against *Leptospira* infection. In the work of Bolin *et al*. [64], vaccination of whole cell-pentavalent vaccine failed to protect cattle from infection with *L*. *borgpetersenii* serovar Hardjo despite high titers of circulating antibodies against LPS. Immunization with whole cell monovalent serovar Hardjo vaccine however, protected cattle from infection, renal colonization, and urinary shedding following *Leptospira* challenge. The protection conferred by this vaccine correlates with its ability to stimulate Th1 response and IFN-γ production [49, 65, 66]. Findings from these studies indicate that anti-LPS humoral immune response is likely to be insufficient for protective immunity in cattle [64, 65, 67], and perhaps in other species. Due to the limited commercial availability of immunological reagents for use in hamsters, we were not able to determine the cell-mediated immune response of hamsters to Lig protein immunizations.

Our results confirm previous studies showing the immunoprotective property of LigA [18–21, 23–26]. Although different serovars and challenge doses were used in these studies, leptospiral challenge was performed through intraperitoneal inoculation. This method allows reproducible amounts of bacteria to be introduced into the animal. However, it does not mimic the natural entry of leptospires into hosts. Future experiments should employ challenge methods that reflect the natural transmission of the pathogen including infecting animals through skin [68, 69] or mucous membranes [57, 65]. Despite the limited number of tools available to measure both humoral and cellular immunity, hamsters remain to be one of the best animal models for use in *Leptospira* vaccine studies owing to their high susceptibility to infection and ability to exhibit clinical features that mimic severe human leptospirosis symptoms [55].

A major challenge in the leptospirosis field is the development of vaccines that confer cross-protection across all pathogenic serovars, protect kidneys from bacterial colonization, and induce long lasting immune protection. An ideal subunit vaccine must have the ability to elicit both humoral and cell-mediated immune responses. Although we have demonstrated the ability of subcutaneous immunization with LigA7’-13 to protect hamsters from lethal infection, renal colonization still persists. In our study, the addition of LigB0-7 did not protect the kidneys from bacterial colonization nor decrease the bacterial load in the renal tubules. However, it is interesting to note that one of the LigB0-7-immunized animals that survived bacterial challenge with sterilizing immunity also generated antibodies recognizing native LigB and cross-reacting with the protective domains of LigA. It may be possible to achieve sterilizing immunity through immunization with a subunit vaccine that generates an immune response that recognizes native epitopes.

## Supporting information

S1 Fig Recognition of purified, recombinant 6xHis-tagged leptospiral protein LipL32 by pooled sera from immunized hamsters. One hundred nanograms per well of purified His-tagged LipL32 were separated in 10% SDS-PAGE then transferred to PVDF membrane for western blot analysis. The membrane was cut to strips and probed with 1:5,000 pooled sera collected before immunization (pre-bleed, P) and at day 32 (last bleed, LB) from immunized and control groups (A). Another membrane strip was incubated with 1:5,000 rabbit α-LipL32 as positive control (Co). As loading controls, the membrane strips were reprobed with 1:5,000 α-LipL32 (B) and 1:1,000 α-6xHis epitope (C). Sera collected from LigA7’-13 and/or LigB0-7 immunized animals do not recognize the unrelated protein nor the epitope tag.
(TIF)

S2 Fig IgG response of hamster groups to recombinant Lig protein immunization. Serum samples were collected from hamsters weekly during the immunization protocol. Anti-LigA7’-13 (A) or anti-LigB0-7 (B) antibody levels were measured in triplicate by ELISA. Each data line represents the average IgG response (minus pre-bleed read) of 5–8 animals over time while error bars indicate standard deviation. Dotted lines indicate vaccine immunization days (blue) or challenge with *Leptospira* (red). There was no difference in the immune response among treatment groups (LigA7’-13, LigB0-7, and LigA7’-13 + LigB0-7) or between the control groups (PBS and PBS + Adjuvant). Anti-LigA and anti-LigB IgG levels of treatment groups were statistically higher compared to control groups starting at day 11 post-immunization (Bonferroni multiple comparison test, *P<0.05, ***P<0.001, ****P<0.0001). Anti-LigB response of LigB-immunized hamsters was statistically higher than LigA7’-13 and LigA7’-13 + LigB0-7 immunized animals at day 11 (^#^*P*<0.05).
(TIF)

S3 Fig *L*. *interrogans* sv. Copenhageni st. Fiocruz L1-130 challenge strain. A 1:50 dilution of *L*. *interrogans* from hamster kidney culture was prepared in liquid EMJH (passage 2), and incubated at 30^°^C at 150 rpm. To determine Lig protein expression, bacteria at an OD_420_ = 0.1 was induced with 120 mM NaCl for 4 h at 30^°^C. Western blot analysis of salt-induced or uninduced *L*. *interrogans* using α-Lig antibody (dilution 1:2,000) show expression of both LigA and LigB by the challenge strain. Membrane was also probed with α-LipL41 (1:10,000 dilution) as loading control.
(TIF)
