# Supplementary figures and images for: Immunoprotective properties of recombinant LigA and LigB in a hamster model of acute leptospirosis
Source: PLoS One. 2017 Jul 13;12(7):e0180004. doi: 10.1371/journal.pone.0180004 (PMC5509140; doi:10.1371/journal.pone.0180004)

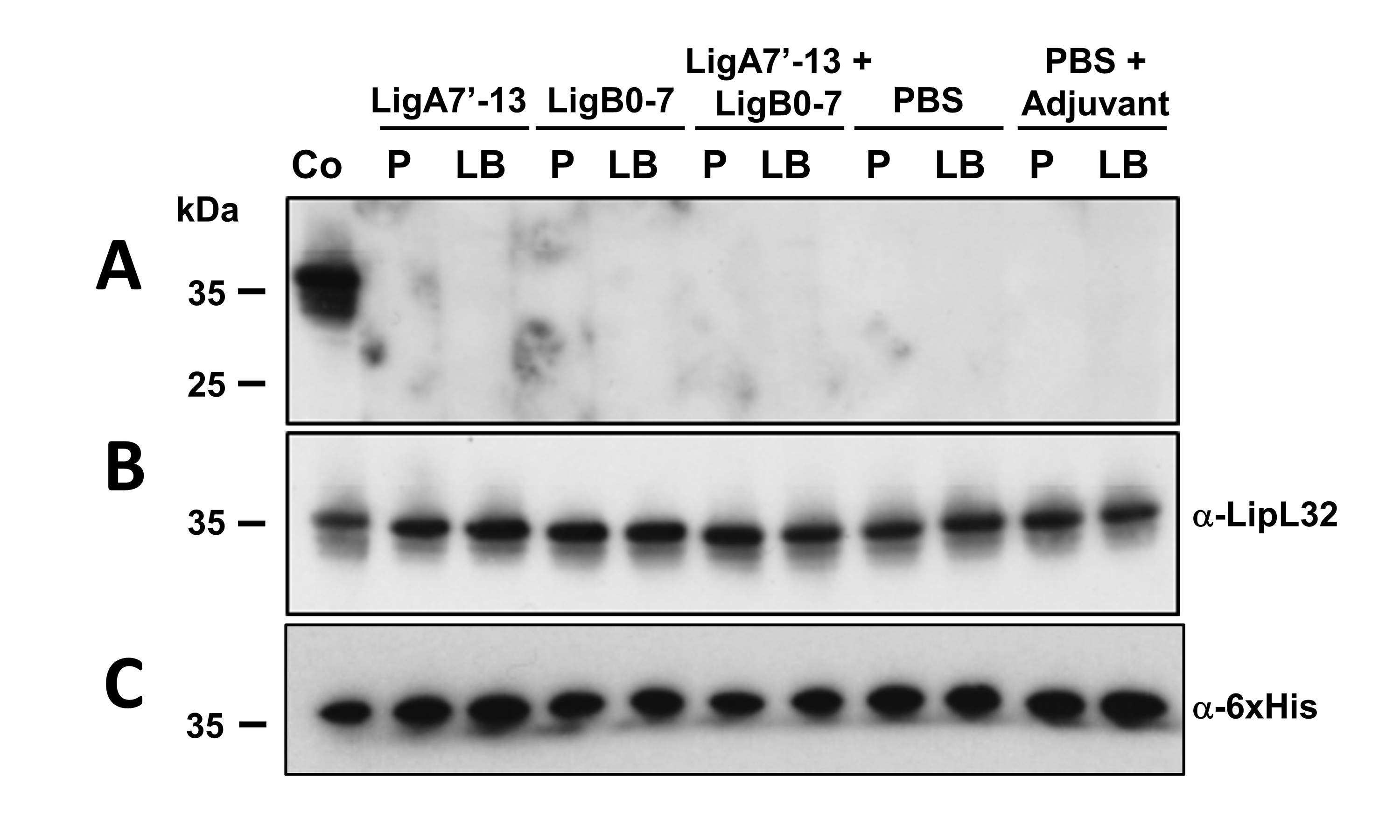

Supplement: S1 Fig — One hundred nanograms per well of purified His-tagged LipL32 were separated in 10% SDS-PAGE then transferred to PVDF membrane for western blot analysis. The membrane was cut to strips and probed with 1:5,000 pooled sera collected before immunization (pre-bleed, P) and at day 32 (last bleed, LB) from immunized and control groups (A). Another membrane strip was incubated with 1:5,000 rabbit α-LipL32 as positive control (Co). As loading controls, the membrane strips were reprobed with 1:5,000 α-LipL32 (B) and 1:1,000 α-6xHis epitope (C). Sera collected from LigA7’-13 and/or LigB0-7 immunized animals do not recognize the unrelated protein nor the epitope tag. (TIF) [file pone.0180004.s001.tif]

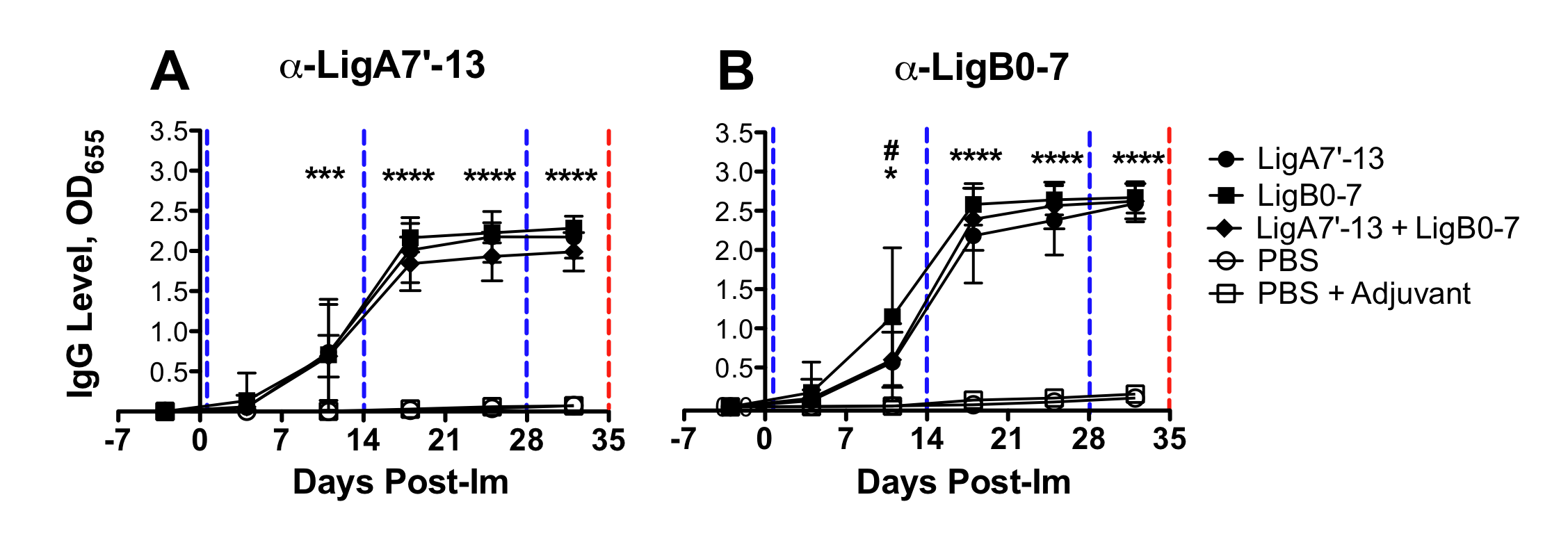

Supplement: S2 Fig — Serum samples were collected from hamsters weekly during the immunization protocol. Anti-LigA7’-13 (A) or anti-LigB0-7 (B) antibody levels were measured in triplicate by ELISA. Each data line represents the average IgG response (minus pre-bleed read) of 5–8 animals over time while error bars indicate standard deviation. Dotted lines indicate vaccine immunization days (blue) or challenge with Leptospira (red). There was no difference in the immune response among treatment groups (LigA7’-13, LigB0-7, and LigA7’-13 + LigB0-7) or between the control groups (PBS and PBS + Adjuvant). Anti-LigA and anti-LigB IgG levels of treatment groups were statistically higher compared to control groups starting at day 11 post-immunization (Bonferroni multiple comparison test, *P<0.05, ***P<0.001, ****P<0.0001). Anti-LigB response of LigB-immunized hamsters was statistically higher than LigA7’-13 and LigA7’-13 + LigB0-7 immunized animals at day 11 (#P<0.05). (TIF) [file pone.0180004.s002.tif]

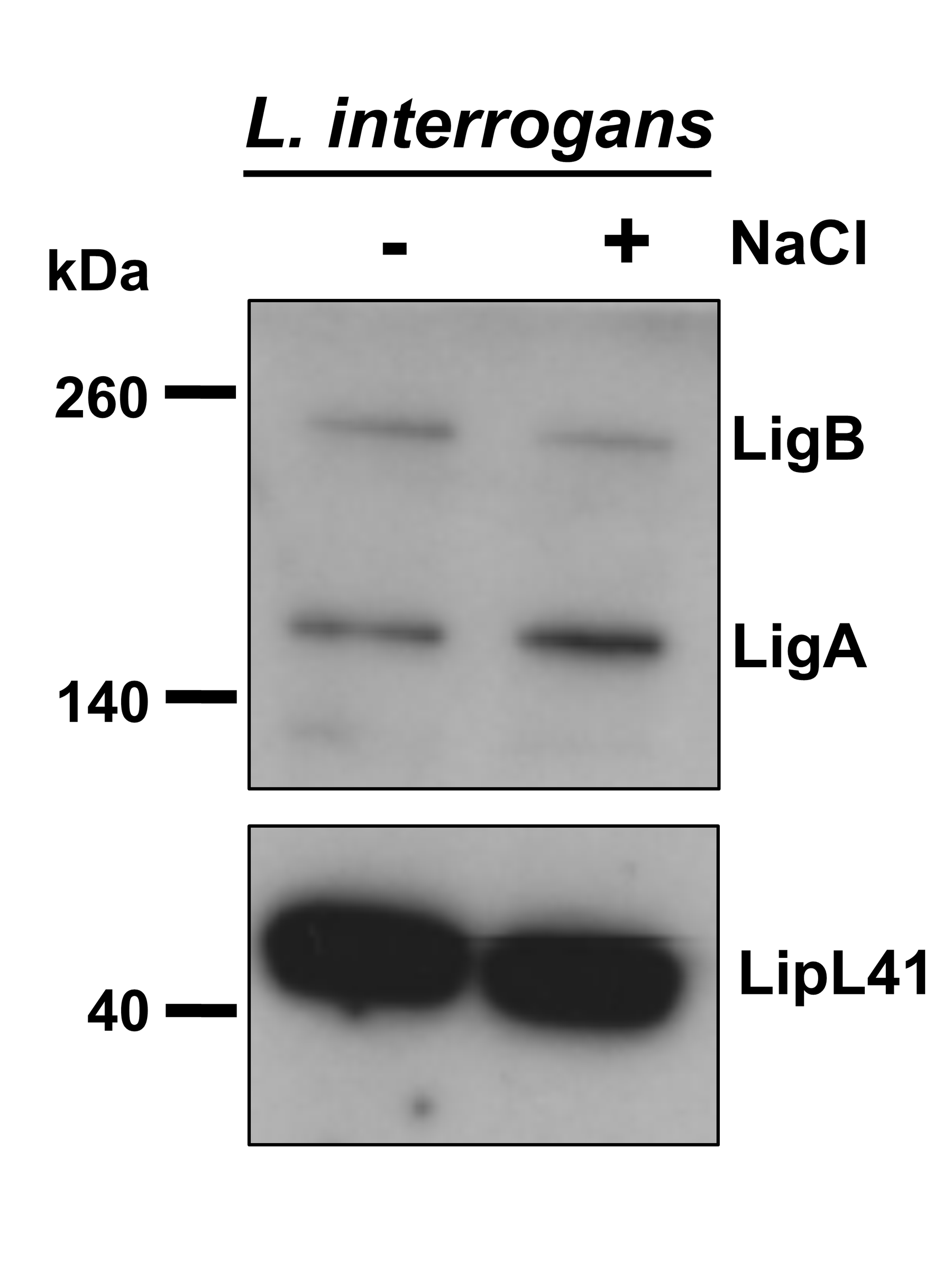

Supplement: S3 Fig — A 1:50 dilution of L. interrogans from hamster kidney culture was prepared in liquid EMJH (passage 2), and incubated at 30°C at 150 rpm. To determine Lig protein expression, bacteria at an OD420 = 0.1 was induced with 120 mM NaCl for 4 h at 30°C. Western blot analysis of salt-induced or uninduced L. interrogans using α-Lig antibody (dilution 1:2,000) show expression of both LigA and LigB by the challenge strain. Membrane was also probed with α-LipL41 (1:10,000 dilution) as loading control. (TIF) [file pone.0180004.s003.tif]
